# Supplementary material for: Widespread but moderate genetic overlap between circulating polyunsaturated fatty acids and brain disorders
Source: J Lipid Res. 2025 Aug 25;66(10):100890. doi: 10.1016/j.jlr.2025.100890 (PMC12492252; doi:10.1016/j.jlr.2025.100890)
Supplement: Supplementary Figures [file mmc1.docx]

**Supplementary Figures**

**
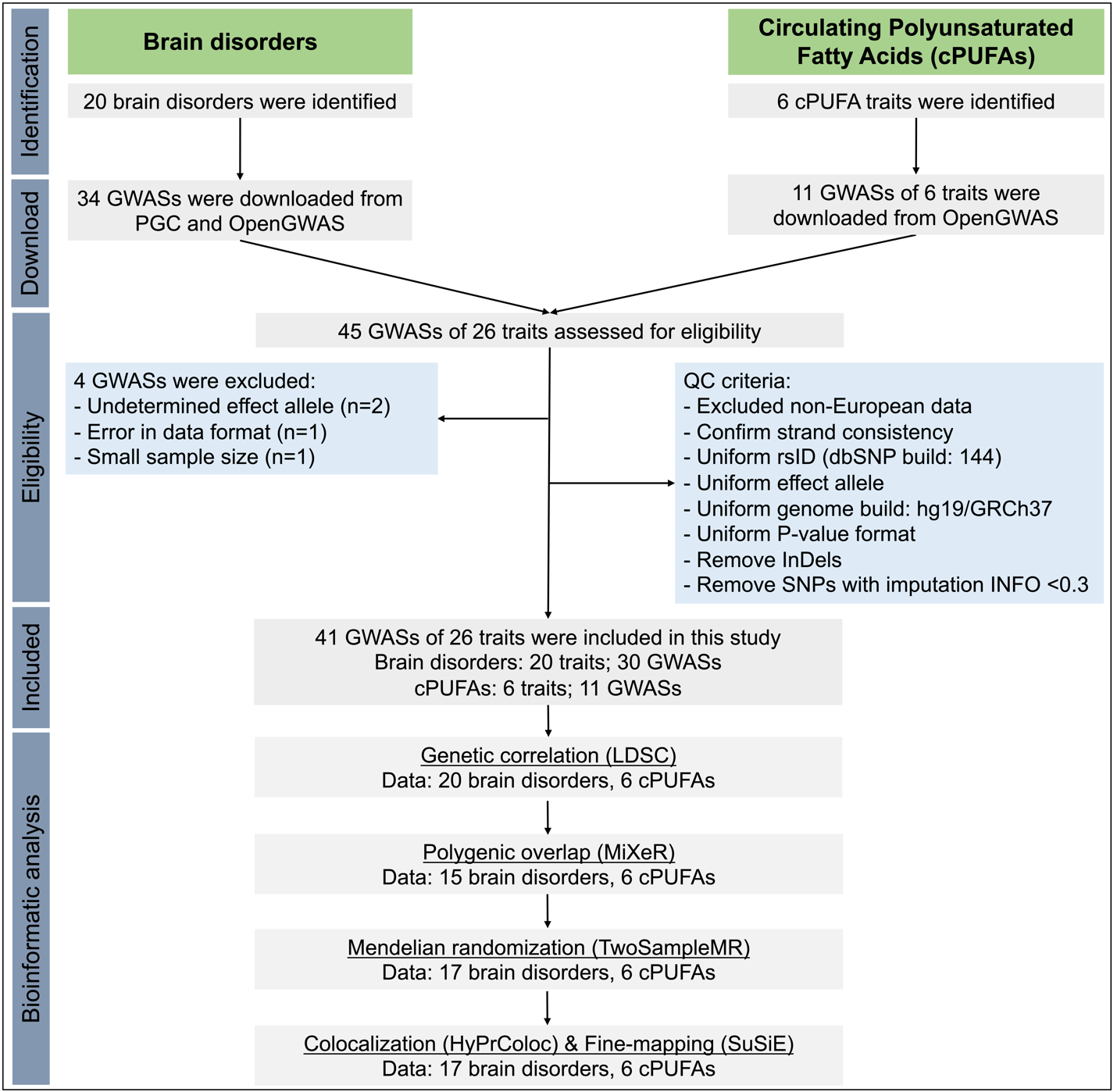
**

**Figure S1.** **Overview of the study.**

**
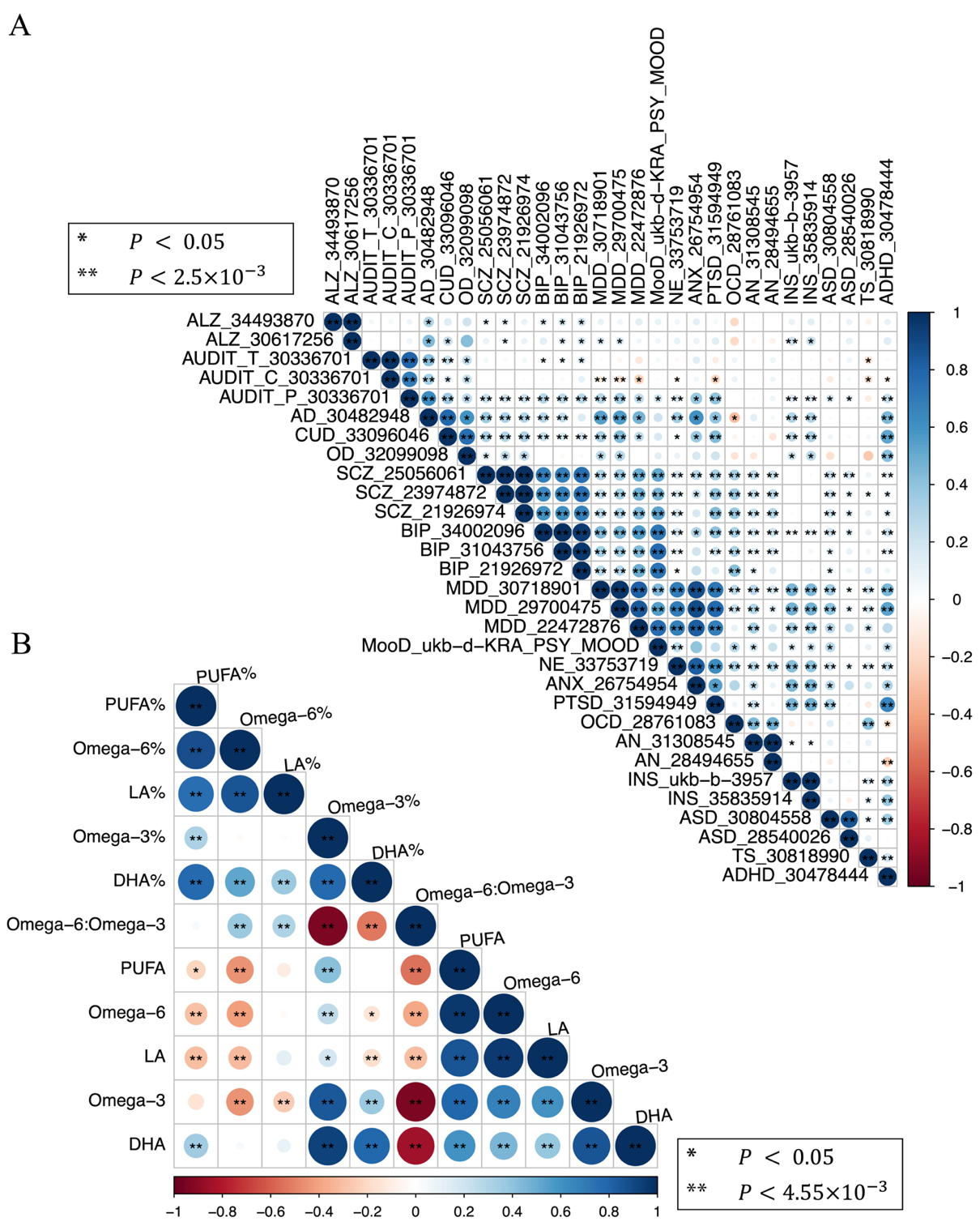
**

**Figure S2. Pairwise genetic correlations A) between brain disorders and B) between cPUFA phenotypes.** GWAS summary statistics of brain disorders are presented in a format with the phenotype_PMID. P-value cutoffs of 0.05, 0.05/# of tests are used to represent different levels of statistical significance; colors are used to represent the degree of genetic correlation (r_g_) between two traits.

**
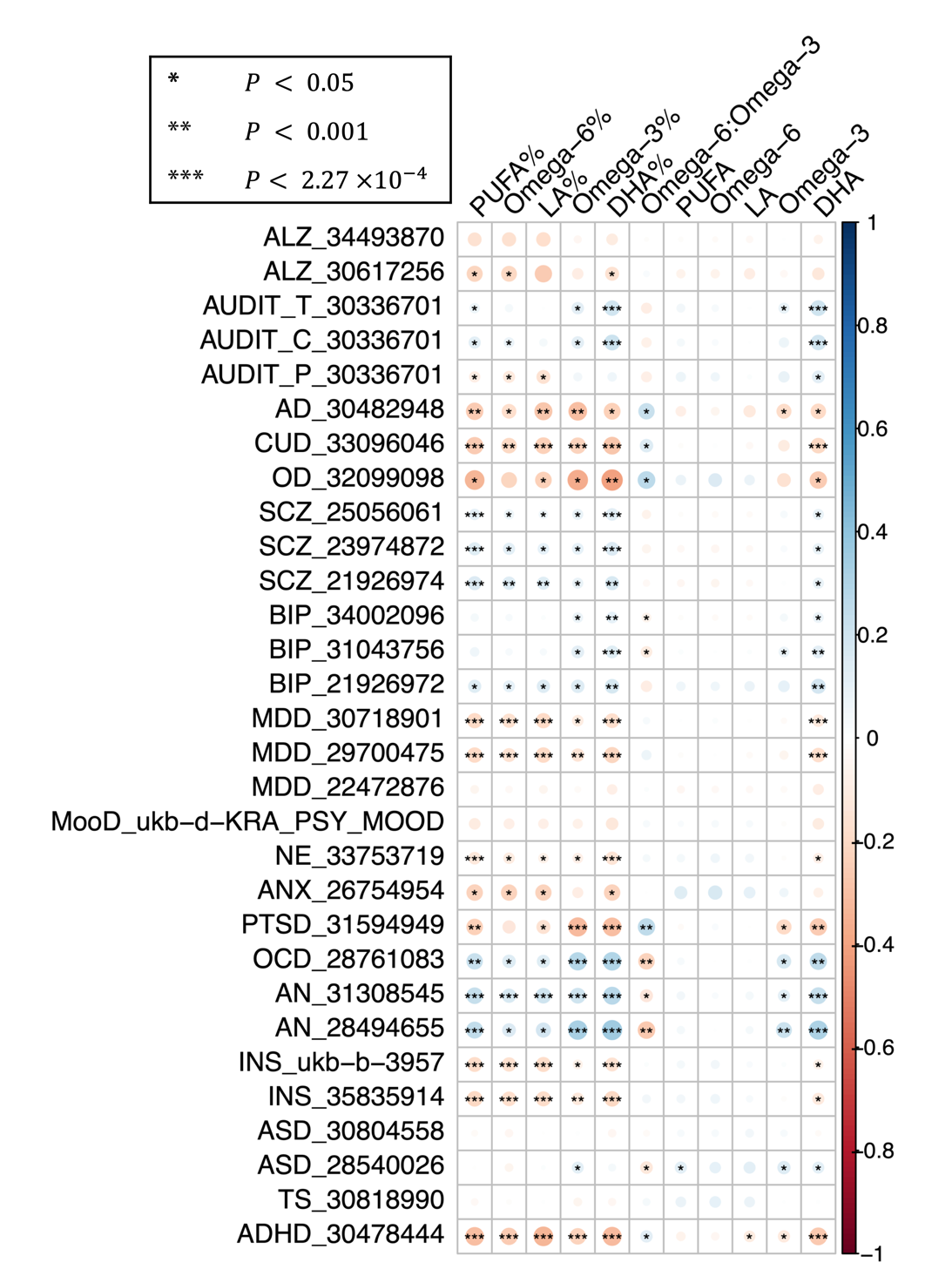
**

**Figure S3. Pairwise genetic correlations between cPUFAs and brain disorders.** P-value cutoffs of 0.05, 0.001, and 4.17×10^-4^ are used to represent different levels of statistical significance; colors are used to represent the degree of genetic correlation (r_g_) between two traits.

**
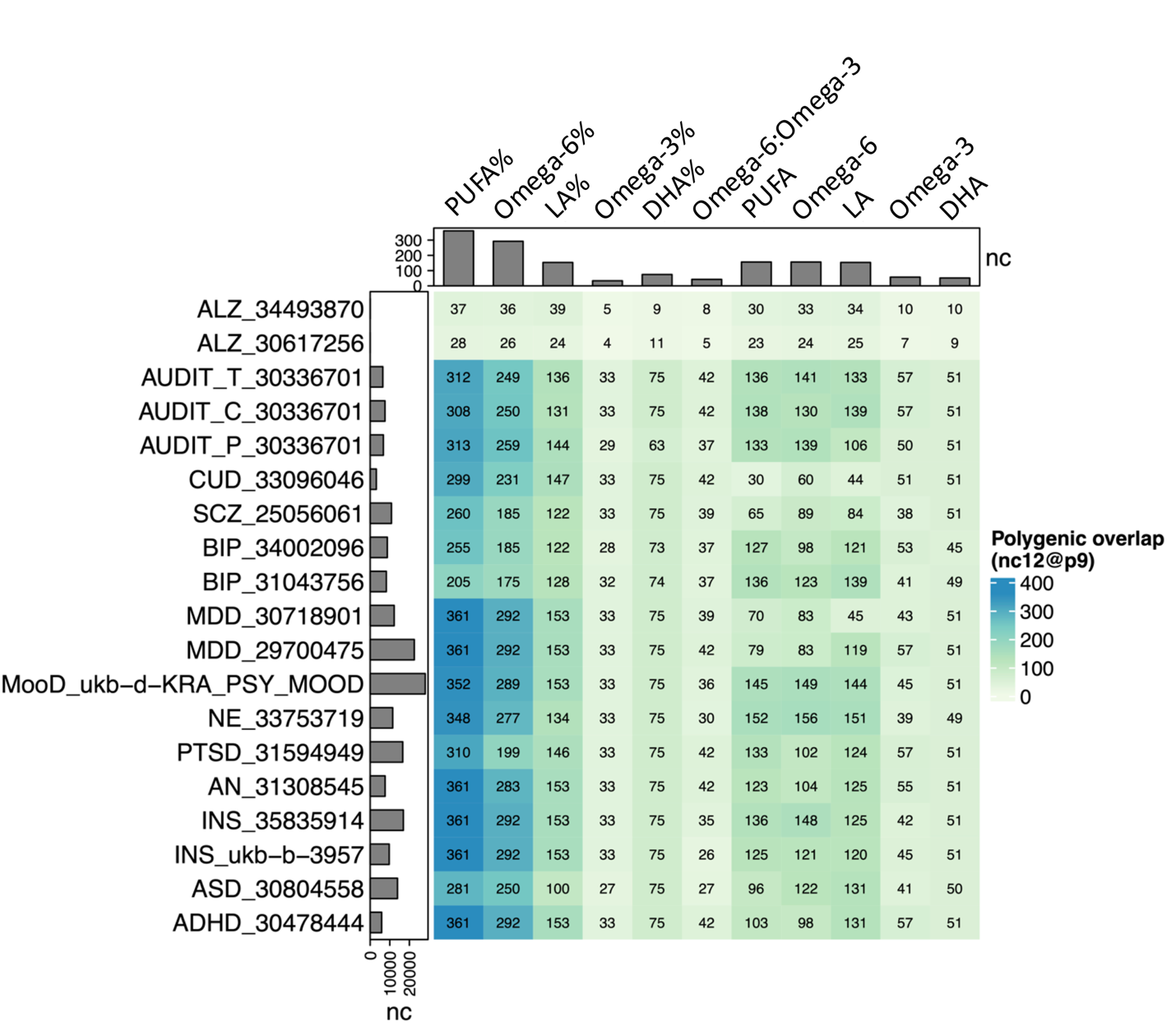
**

**Figure S4. Pairwise polygenic overlaps between cPUFAs and brain disorders.** The color and number of each box indicate the degree of polygenic overlap and the number of causally associated SNPs shared between cPUFAs and brain disorders (nc_12_). Barplots on the top and left indicate the number of cPUFAs- and brain disorders- associated variants, respectively, which explain 90% of SNP-based heritability.

**
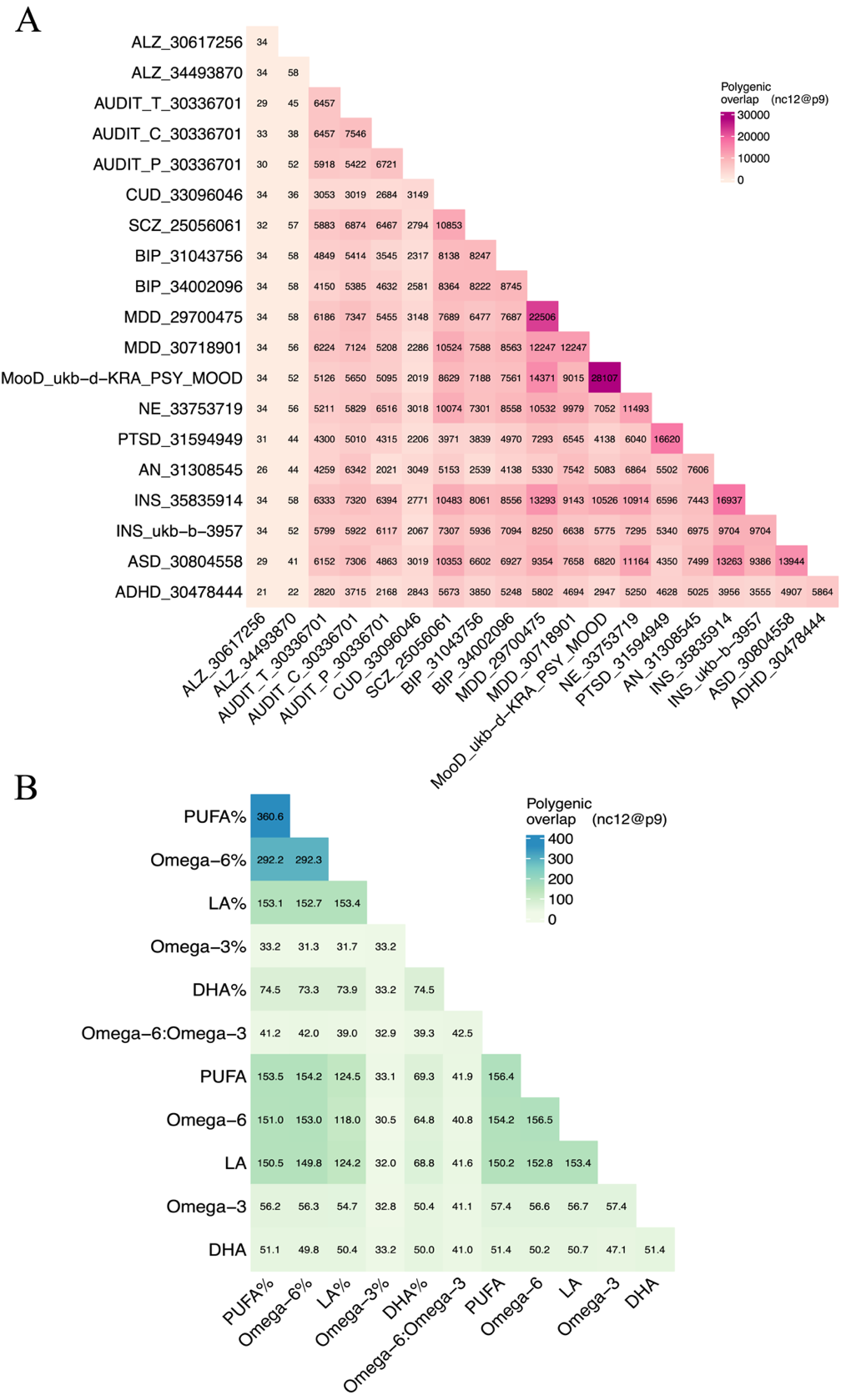
**

**Figure S5. Pairwise polygenic overlaps** **A) between brain disorders and B) between cPUFAs.** The color and number of each box indicate the degree of polygenic overlap and the number of causally associated SNPs that explain 90% of SNP-based heritability, shared between cPUFAs or between brain disorders (nc_12_).

**
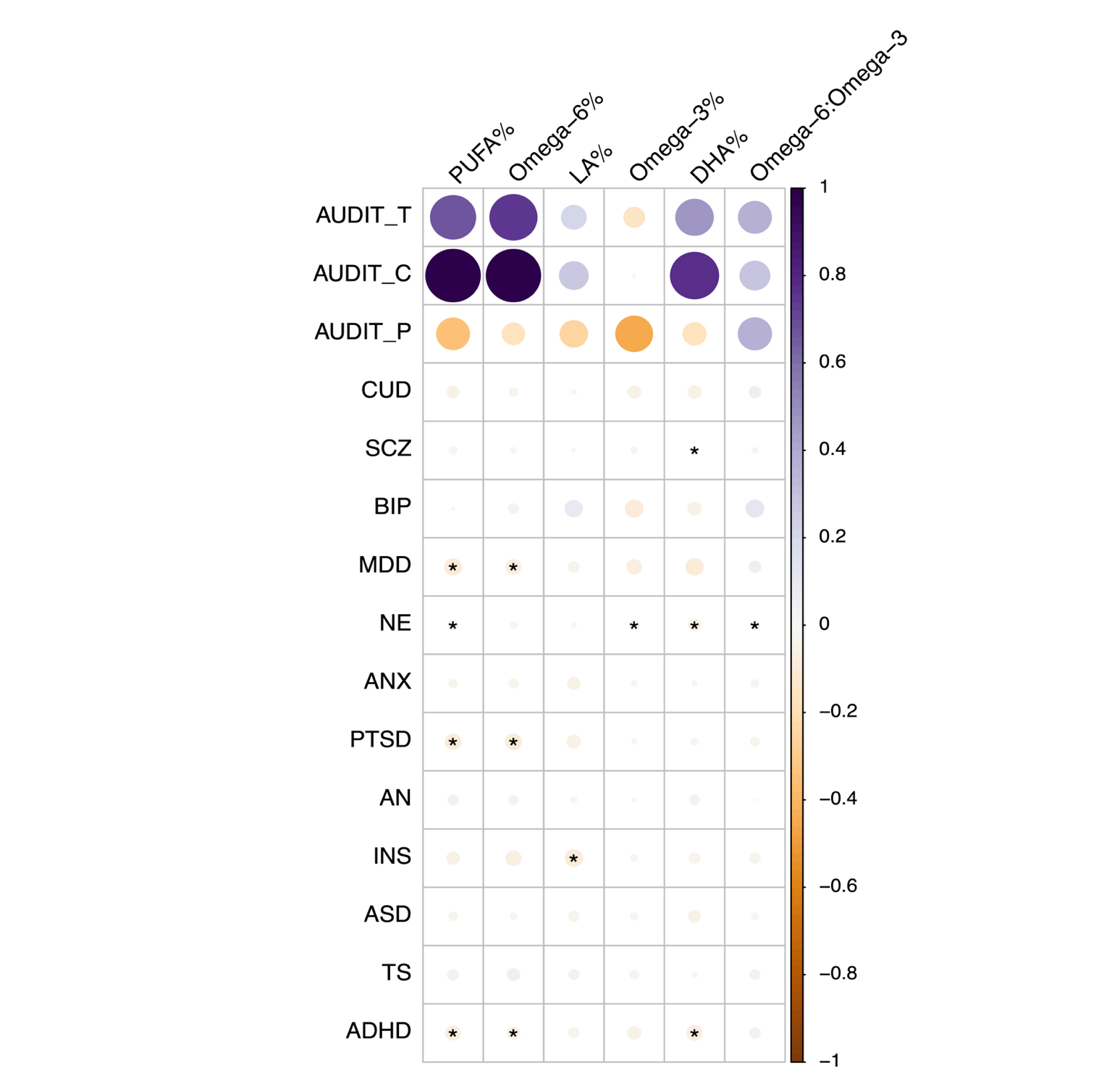
**

**Figure S6. Reverse MR results.** Heatmap summarized the causal effects of 15 brain disorders on six cPUFAs. Except for four brain disorders (ANX, ASD, PTSD, TS), an IVW *P* value of 0.05 is used to represent statistical significance; colors are used to represent the causal effects (β_IVW_) of cPUFAs on brain disorders. The causal effect of four brain disorders (ANX, ASD, PTSD, TS) on cPUFAs and corresponding *P* values were estimated using the Wald ratio test.

**
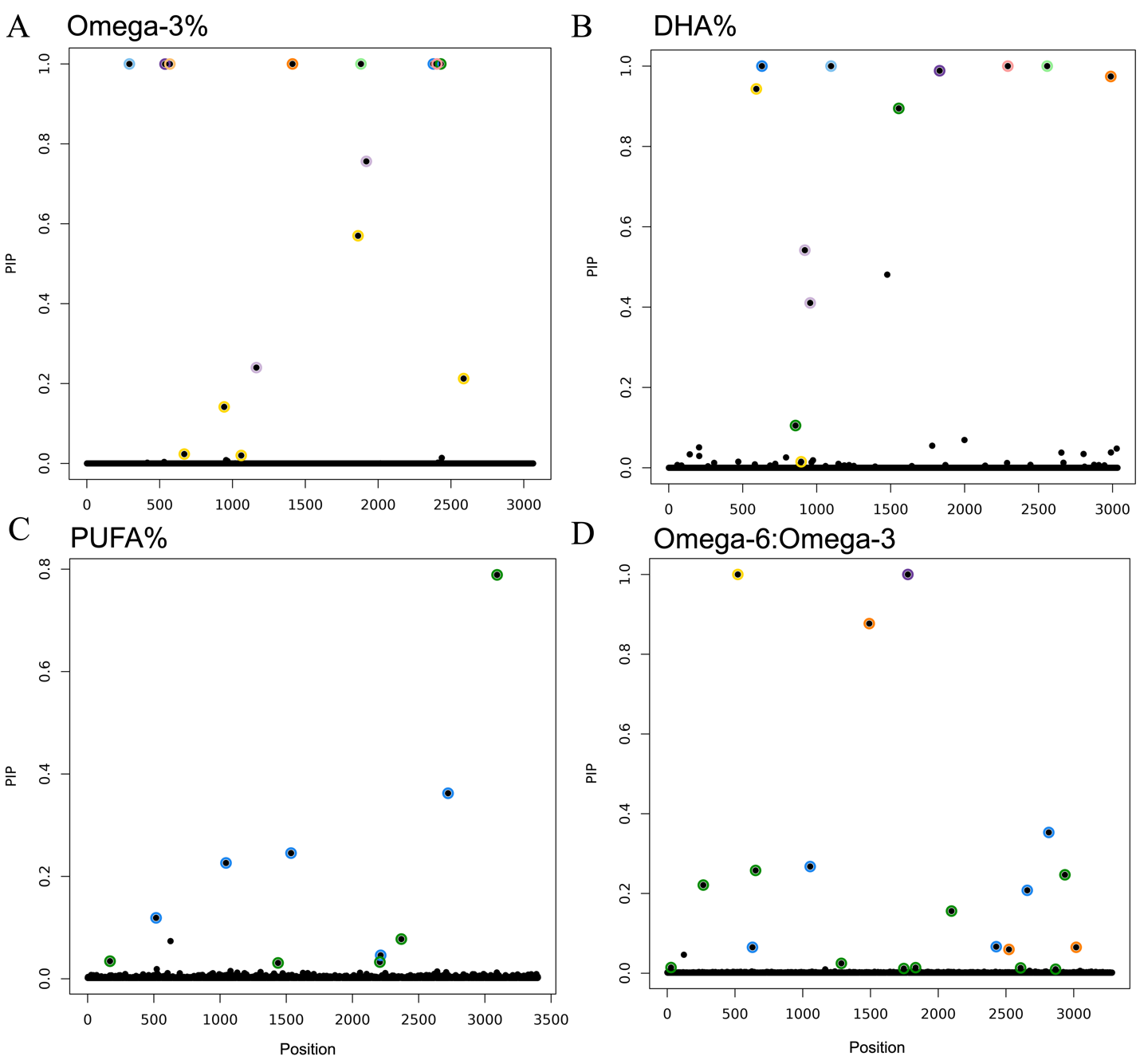
**

**Figure S7. Statistical fine-mapping posterior inclusion probability plots in 11q12.1-11q12.3 for A) omega-3%, B) DHA%, C) PUFA% and D) omega-6:omega-3.** The variant position is shown on the x-axis, with posterior inclusion probability (PIP) on the y-axis. Colors are used to represent different 95% credible sets. Each 95% credible set contains a causal variant. There are multiple putative causal variants in the region for omega-3%, DHA%, PUFA%, and omega-6:omega-3.

**
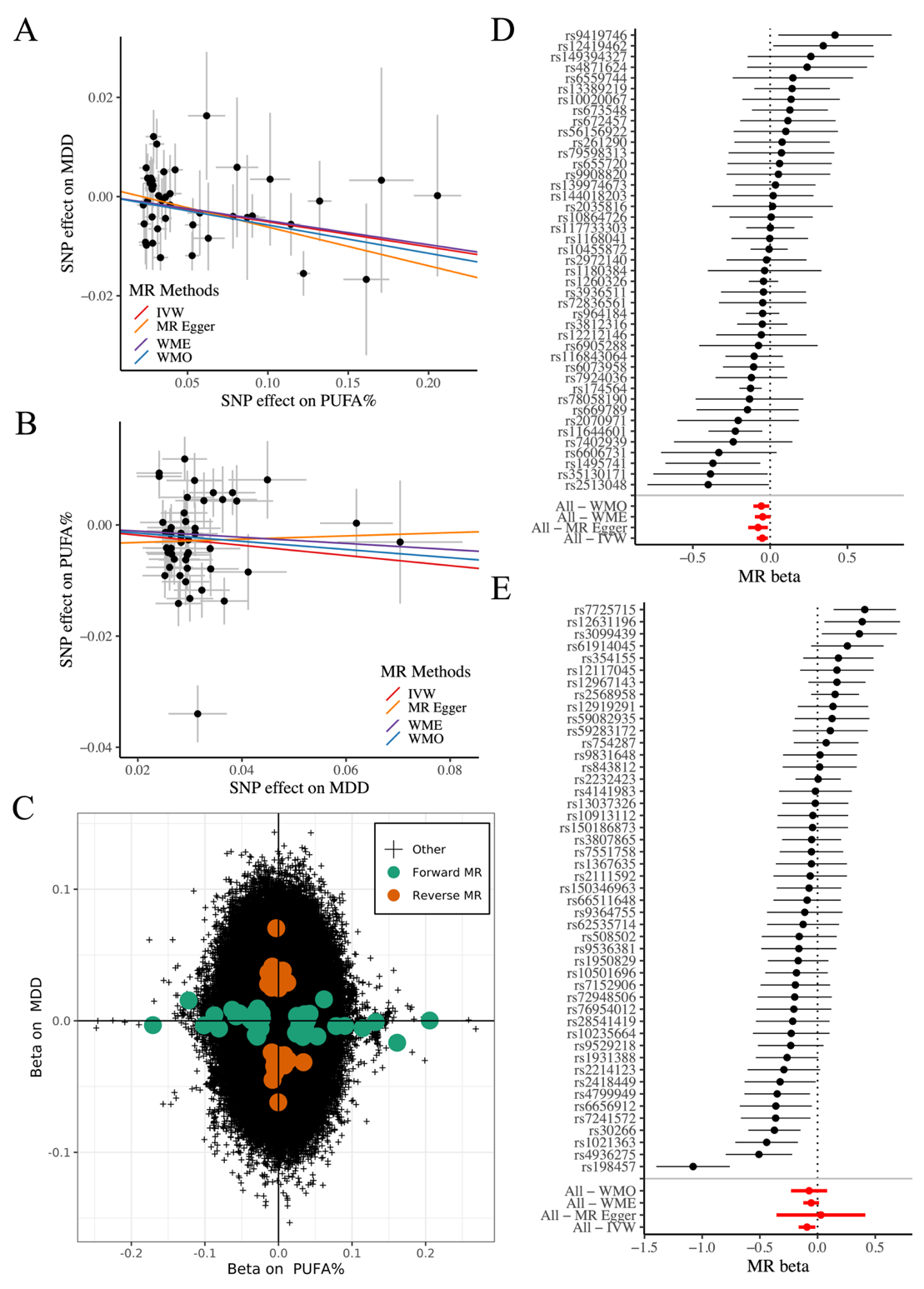
**

**Figure S8. Bidirectional association between PUFA% and MDD. A)** Forward MR estimated causal effects of PUFA% (x-axis) on MDD (y-axis). Causal effects estimated by the four models are shown by fitted lines; slopes of these lines indicate causal effect sizes. **B)** Reverse MR estimated causal effects of MDD (x-axis) on PUFA% (y-axis). **C)** Genetic effect of genome-wide SNPs on PUFA% (x-axis) and MDD. Genetic variants selected in forward and reverse MR analysis are shown in green and red, respectively. **D)** The forest plot shows 43 genetic instruments included in the PUFA%-MDD forward MR analysis. The causal effects estimated by the four models are marked in red. **E)** The forest plot shows 48 genetic instruments included in the MDD-PUFA% reverse MR analysis.

**
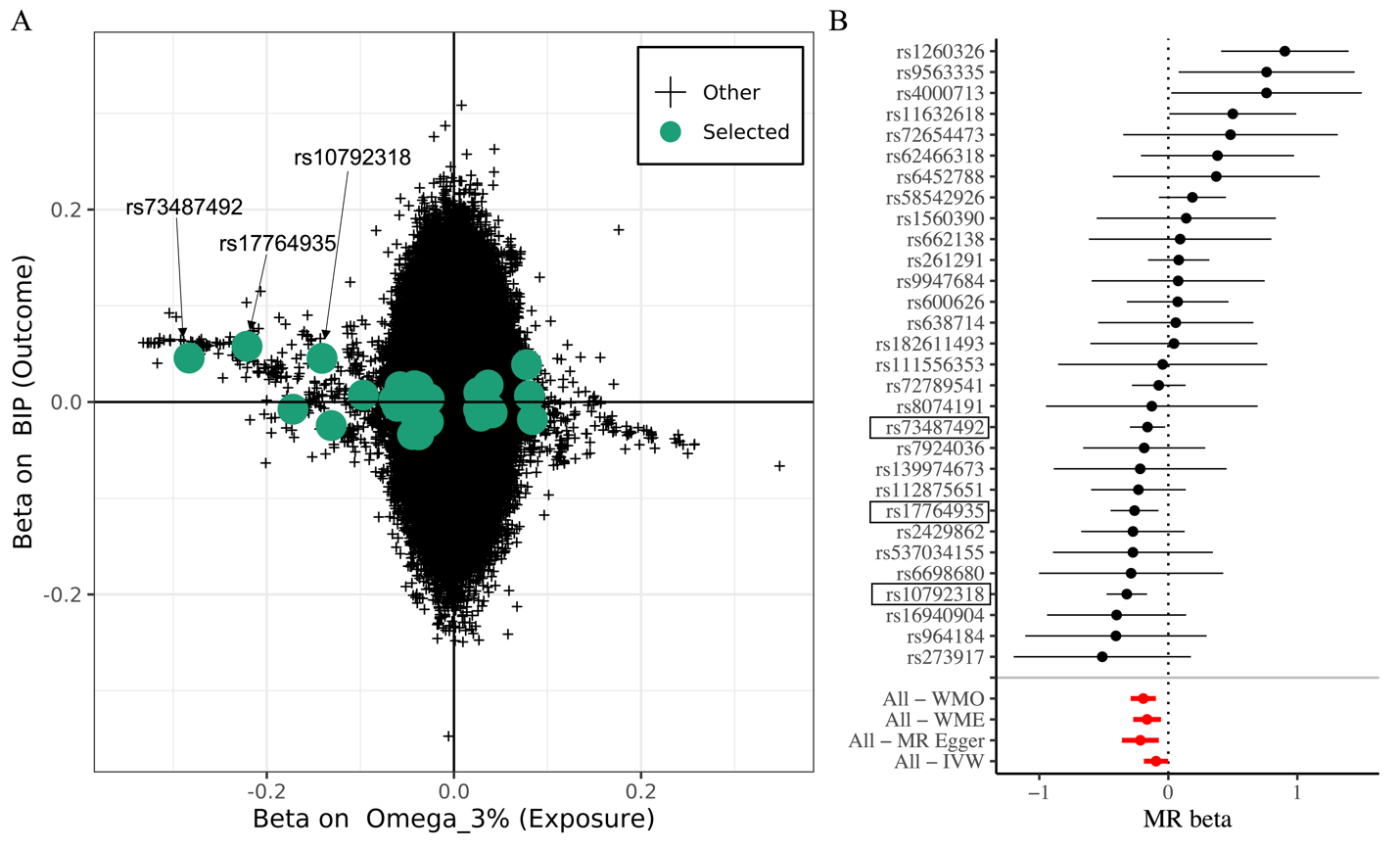
**

**Figure S9.** **Differences in the SNP set in omega-3%-BIP genetic correlation and MR analysis. A)** Genetic effect of genome-wide SNPs included in genetic correlation analysis on omega-3% (x-axis) and BIP (y-axis). Genetic variants that are selected in omega-3%-BIP MR analysis are shown in green. Three highlighted SNPs are in the FADS locus. **B)** The forest plot shows 30 genetic instruments included in the omega-3%-BIP MR analysis. The causal effects estimated by the four models are marked in red.
